# Harnessing adaptation has increased French apple yields but heat stress mitigation will become essential by 2050

**DOI:** 10.1038/s43016-026-01333-0

**Published:** 2026-03-31

**Authors:** Laurent Giguère, Rachael D. Garrett, Matthieu Stigler, Graham J. Dow

**Affiliations:** 1 Department of Environmental Systems Science, ETH Zürich, Zurich, Switzerland; 2 Department of Geography, Conservation Research Institute, University of Cambridge, Cambridge, UK; 3 Crop Science and Production Systems, NIAB, Cambridge, UK

**Keywords:** Agriculture, Plant physiology, Climate-change impacts, Climate-change adaptation

## Abstract

Evidence on how climate change affects perennial fruit crops remains limited. Here, focusing on historical apple production in France from 1989 to 2020, we find that most regions adapted to harness warming-related benefits, especially in the apple blooming period. However, in hotter Mediterranean departments, adaptations to mitigate yield losses were crucial to avoiding greater losses. Although historical effects were mostly positive due to ‘harnessing adaptation’, temperatures will increasingly exceed optimal thresholds, increasing the number of regions experiencing net losses by 2050 unless heat stress mitigation improves.

## Abstract (web summary)

French apple yields have increased as temperatures have shifted towards yield optima, but by 2050, more regions may experience yield losses unless heat stress mitigation improves.

## Main

Understanding the impact of climate change on food production is key to finding new strategies for ensuring the food security of future generations and the resilience of farming communities. So far, many studies have investigated this question for widely grown annual crops such as maize, soybeans and wheat^1–5^. Studies on annuals include understanding the potential of long-run adaptation to mitigate the negative impacts of temperature increases on yields^6–10^. However, studies examining the effects of climate change on the yields of perennial crops—particularly fruit trees—are less common in the literature.

To our knowledge, no study has quantified the adaptation potential of perennial fruit crops to climate change, despite their considerable economic and nutritional importance globally. This knowledge gap is worrisome as perennials may be more heavily impacted by climate change because their adaptation options are more limited. While some risks can be mitigated by management of the existing trees (for example, through changes to irrigation or thinning practices), renewal of trees through new cultivars may be necessary to reduce certain risks or take advantage of future changes. The high costs of tree replanting and delays before reaching full production usually result in commercial fruit trees being kept for 15–20 years. This economic reality may constrain short-term adaptation to new climatic conditions.

To study the response of perennial fruit crops to climate change, while considering their capacity to adapt, we analyse the case of apples. Apples are the most widely grown perennial crop for fresh consumption in temperate climates^11^ and a major contributor to daily intake of essential vitamins and dietary fibres. Among the studies of climate impacts on the yields of perennial crops^12–15^, only one has focused on apples^12^; it neither examines impacts at a macroscale nor considers the role of adaptation—gaps that the current study aims to address.

In this Brief Communication, we investigate how climate change affects apple production and how apple-growing systems can adapt to climate change following a two-step approach. First, we looked at the effect of past climate trends on apple yields using panel regression techniques and quantified how much of this effect was conditional on climate adaptation taking place. Second, we used our estimates from the regression to forecast the impact of future climate change on apple yields, under scenarios of full and zero adaptation.

We analysed adaptation as the difference between the impact on apple yields of changes in weather conditions observable in the short run and the same changes happening over the long run based on the theoretical framework recently developed by Mérel and Gammans^8^. Such adaptation within growing areas involves management changes and technological innovations implemented over many years. In line with the IPCC’s definition of climate adaptation: “…the process of adjustment to actual or expected climate and its effects in order to moderate harm or take advantage of beneficial opportunities”^16^, we provide a conceptual advancement to the existing literature by distinguishing between two types of adaptation. ‘Harnessing adaptation’, meant to improve yield, is an adaptation taking place when temperatures increase beyond the historical mean and the historical mean lies below the long-run optimum. ‘Mitigating adaptation’, meant to dampen yield losses, takes place when temperatures increase beyond the historical mean and the historical mean lies above the long-run optimum or in contexts where no such optimal temperature has been established (see equations (4) and (5) in the Methods). Finally, unlike previous studies seeking to measure climate adaptation, we examined whether there is evidence of geographic relocation as an additional form of climate adaptation^17^.

Our analysis focuses on France, the world’s ninth-largest apple producer in 2022 (ref. ^11^). France is an ideal case study of climate change’s effects on apple production given the high heterogeneity of climates where apples are grown. We estimated our model using yield data provided by the French Ministry of Agriculture for 32 French departments over the period extending from 1989 to 2020 and gridded weather data from the EU Joint Research Centre’s AGRI4CAST Toolbox. Yields were measured in tonnes per hectare and included all apples harvested, with no distinction between varieties and quality grades. Even though different apple varieties may be affected differently by changes in temperature and precipitation patterns, we chose to bundle them all together in our analysis for two reasons. First, running separate regression analyses for different cultivars would have excluded any potential change in varietal mix from growers’ long-run response to changes in climate, thereby forcing our model to ignore the crucial role that varietal choices might have played over the years in helping them either harness or mitigate the effects of climate change on yields. Second, production data on varieties grown in France are very limited in scope (Supplementary Fig. 2). All we know is that, from 1989 until 2020, the share of Golden Delicious apples at the national level has gone from 54% down to 28% of total area grown, Granny Smith stayed around 10%, and the share of other cultivars (including Gala) increased from 36% to 65%. There is no information on Gala before 2016, after which we know it makes up 19% of the area. Although it is possible that some of the shifts away from Golden Delicious are due to climate, the most likely reason is shifting consumption patterns. New varieties in the market in Europe tend to have improved texture and acidity relative to Golden Delicious, making it the least favourite in consumer preference studies^18^. Other more specific shifts in cultivars are unclear given relatively stable levels for Granny and Gala, and the lack of distinction in the other varieties. These trends and data deficiencies substantiate our approach to bundle all varieties. Finally, because apple fruit growth responds differently to temperature^19–21^ and water availability^22,23^ in each of the apple fruit’s phenological phases, our econometric model required the inclusion of those stages (blooming phase, early growth/cell division phase, late growth/cell expansion phase and harvest phase), which constitutes an innovation from previous studies focusing on climate adaptation.

**Fig. 1:**
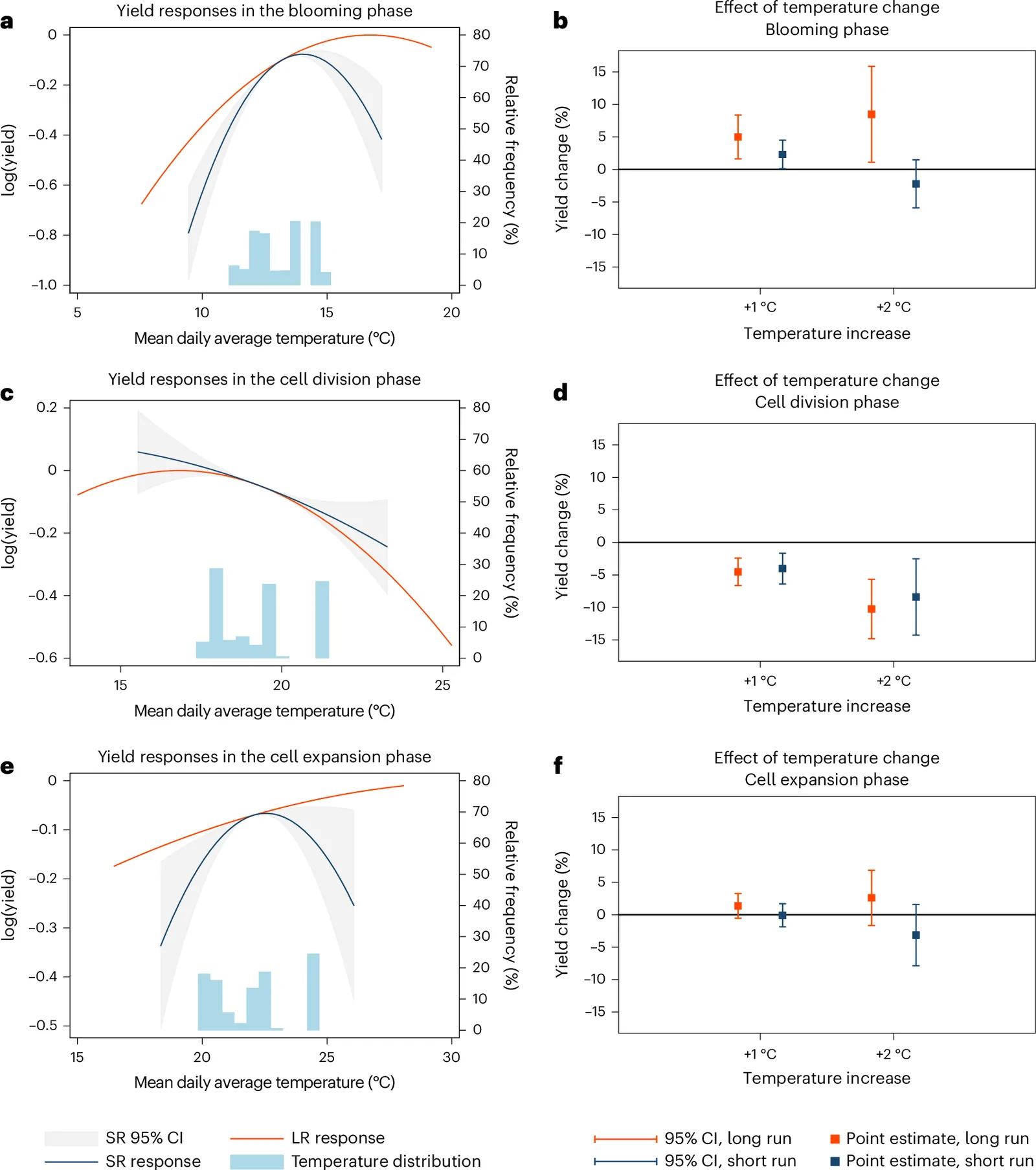
Short- and long-run temperature–yield relationships in French apple-growing regions. **a**,**c**,**e**, The concave temperature–yield relationship for the blooming (**a**), cell division (**c**) and cell expansion (**e**) phases of the season together with the cross-departmental distribution of mean temperatures over the same phase underneath. The difference between the short-run (SR) and long-run (LR) response curves, which are tangent at the 2001–2020 surface-weighted national mean, represents the effect of adaptation on yields. The optimum is absent in **e** because it is statistically insignificant and out of reach. **b**,**d**,**f**, Estimated long-run and short-run yield changes (%) following a 1 °C and 2 °C deviation from the 2001–2020 surface-weighted national mean for the blooming (**b**), cell division (**d**) and cell expansion (**f**) phases. confidence intervals are computed using the delta method. All estimates used in the figure are derived from a panel regression (Methods) with sample size *n* = 1,024. CI, confidence interval. Source data

**Fig. 2:**
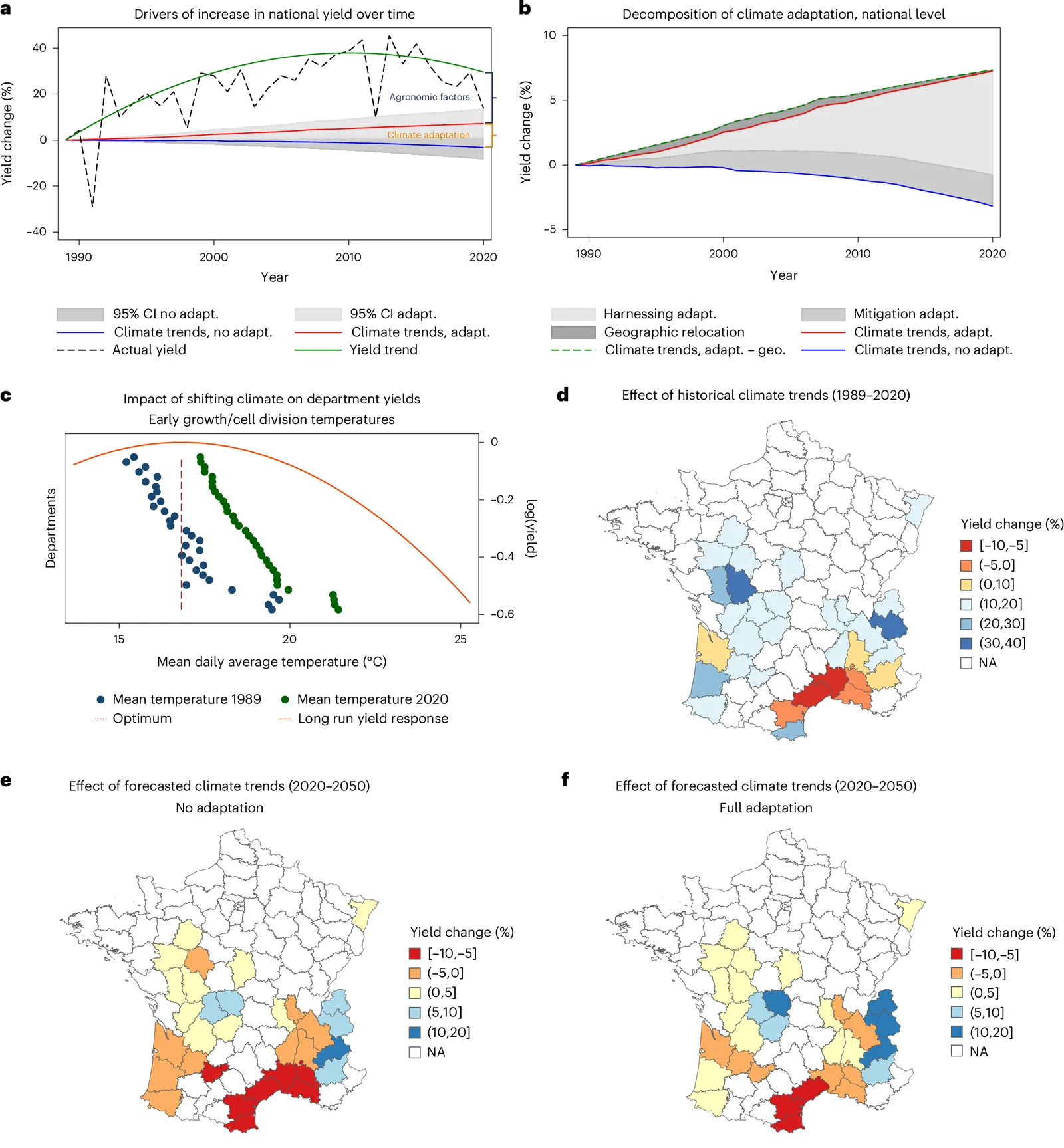
Effect of historical and future climate trends on French apple yields. **a**, National yields as a function of combined climate trends over the sample period, with and without adaptation (adapt.), compared with the actual observed yield trend. Its confidence intervals are computed using the delta method. **b**, Zoom-in on the portion of **a** corresponding to the effect of climate adaptation on the yield response, showing the counterfactual yield response—assuming adaptation occurred without geographic relocation (dashed green line). **c**, Departmental mean temperatures in 1989 and 2020 with respect to the long-run yield response taken from Fig. 1c for the early growth phase. **d**, Changes in departmental yields caused by historical climate trends at the local level assuming full adaptation. **e**,**f**, Forecasted yield changes at the department level assuming zero (**e**) and full (**f**) adaptation respectively under the RCP 4.5 scenario. All estimates used in the figure are derived from a panel regression (Methods) with sample size *n* = 1,024. Basemaps in **d**–**f** from GADM version 2.5 (https://www.gadm.org). NA, non-assessed. Source data

Consistent with the ecophysiological literature, our results show that, besides the occasional late frost, apple yields are mostly influenced by changes in temperature in the first two phenological phases of the season (Supplementary Table 1). Conversely, precipitation does not play any significant role throughout the growing season, probably because all areas studied are heavily irrigated (Supplementary Table 3). In 2020, all apple-growing departments had mean temperatures below the long-run optimum during the blooming phase (Fig. 1a) and above the long-run optimum during the cell division phase (Figs. 1c and 2c). Short-term optimal temperatures in 2020 (Supplementary Table 8), which differ from the long-term optimum when adaptation is possible (Supplementary Methods section 10) and thus pertain only to the blooming phase, are location-dependent but consistently above the historical mean for all departments, given the shape of the temperature–yield relationship during this phase (Supplementary Methods section 11). In fact, during the historical period, 87% of all recorded positive temperature shocks (that is, positive deviations from the local mean) across all departments had a beneficial impact on yields (+2.21% on average) according to our model estimates. The remaining shocks had a negative impact on yields given their correlation with spikes in daily temperature reaching beyond physiological, that is, long-run optimal temperatures. The difference between short-term and long-term optimal temperatures in the blooming phase is well illustrated in Fig. 1b, which shows that a 1 °C temperature increase from the 2020 national mean causes a yield rise both in the short and long run (+2.35% and +5.05%, respectively), while a 2 °C temperature increase does so only in the long run.

All empirical temperature optima derived from our statistical model (Supplementary Table 8) are in line with the biological optima known from the plant physiology literature^21,24–26^. Based on the distribution of local means in 2020 relative to these optima, from 1989 until 2020, long-term increases in temperature during the blooming phase have propelled average yields upwards in all French regions, whereas rising temperatures during the cell division phase have had either a neutral or negative effect, depending on the department (Fig. 2c). As seen in Fig. 1a,b, harnessing adaptation was key to raising yields in the blooming phase, while no adaptation was observed in the cell division phase (Fig. 1c,d). Because there are no data available on exactly how apple producers adapt in the orchards themselves, we hypothesize that they have gradually adjusted their training systems and thinning methods and adopted higher-yielding cultivars that thrive within those systems. Further study is needed to identify the specific pathways through which harnessing adaptation takes place.

Temperatures during the cell expansion phase do not influence yields in the long run—that is, in adapted systems (Supplementary Table 1)—but mitigation adaptation occurring in this phase can potentially eliminate all long-term yield losses (Fig. 1f and 2b), should they reach statistically significant levels. Still, the effect of mitigation adaptation is small compared with the harnessing adaptation happening during blooming (+1.4% versus +2.6% for any 1 °C temperature deviation from the mean). Irrigation probably acts as an effective buffer against heat stress in this phase, as opposed to the cell division phase, which is known to be more sensitive^20,21,27^.

National apple yields have gone up by 29.5% from 1989 until 2020, 7.2% of which was the result of production systems benefitting from climate trends through adaptation, while the rest of the increase was caused by unobservable agronomic improvements not tied to climate adaptation such as increased orchard density and the adoption of higher-yielding cultivars for commercial purposes (Fig. 2a). Without climate adaptation, climate trends would have instead led to a 3.2% drop in national yields (Fig. 2a,b). Most of the adaptation was of the harnessing type and occurred during the blooming phase, while the remainder was mitigating adaptation, which prevented yield losses and took place during the cell expansion phase (Fig. 2b).

Aggregate yield benefits at the national level overshadow important disparities between the different growing regions (Fig. 2d). Northern and high-altitude departments have largely benefitted from changing climate conditions, with yields in some regions increasing by more than 30% due to harnessing adaptation, and, to a lesser extent, from downward trends in late frost. Conversely, Mediterranean departments have suffered small losses ranging between 1% and 7% as a result of rising temperatures in the cell division phase, the negative effects of which could not be mitigated.

In the next decades of climate change leading up to 2050, regional disparities are expected to carry forward. Under a scenario of partial reduction in GHG emissions (Representative Concentration Pathway 4.5, RCP 4.5), our results indicate diminishing yield gains and rising yield losses from higher heat stress in the cell division phase. Even if growing systems across all of France fully harness the potential remaining benefits from climate change in this scenario, the number of departments expected to see their yields decrease goes from 5 to 11 (Fig. 2f). In the most pessimistic scenario, in which producers fail to harness any climate benefits, this number rises to 16 departments (Fig. 2e), although national yields decline by only 0.44%. The results do not differ much under the RCP 8.5 business-as-usual scenario (Supplementary Fig. 1).

In light of these expectations, future adaptation strategies will be needed to protect apple yields against heat stress caused by climate change. Geographic relocation is one potential strategy, but there is no evidence that production relocation has helped to avoid climate-change-induced losses in the recent past (Fig. 2b, Supplementary Methods section 6 and Supplementary Table 7). The most promising route for adaptation is likely to be the adoption of heat-tolerant cultivars, examples of which have already been developed in some of the hottest and driest apple-growing regions, such as Spain and Israel. In the short term, orchard systems that manage canopy architecture to enable efficient and time-sensitive shading of apple trees could potentially reduce phenological heat stress^28,29^. Estimating the costs of these adaptation strategies, and thereby their economic feasibility in various apple-growing regions, represents a promising avenue for future research.

## Methods

### Ethical compliance statement

The study protocol underlying the current work did not need any formal approval from an ethics board or committee given the nature of both the research conducted and the data used (publicly available data with no restrictions on their use and diffusion). As such, it complies with all relevant ethical regulations.

### Model estimation

To obtain the historical yield–weather relationship needed for our analysis, we estimated a fixed-effect regression based on the functional form found in Mérel and Gammans^8^, as shown here:

1 $${y}_{{it}}={\mathbf{\upbeta}}_{\mathbf{1}}{{\bf{x}}}_{{it}}+{\mathbf{\upbeta}}_{\mathbf{2}}{{\bf{x}}}_{{it}}^{2}+{\mathbf{\upbeta}}_{\mathbf{3}}{\left({{\bf{x}}}_{{it}}-{{\mathbf{\upmu }}}_{{it}}\right)}^{2}+{{\gamma }_{j}t+\delta }_{j}{t}^{2}+{\alpha }_{i}+{\varepsilon }_{{it}},$$

where ${y}_{{it}}$ represents the log of annual yields adjusted for biennial bearing using AR(1) regression as a first stage (Supplementary Methods section 8) in year *t* and in department *i*, ${{\bf{x}}}_{{it}}$ is a vector of yearly weather variables, **β**_**123**_ are row vectors of the coefficients tied to each weather variable, ${{\bf{\upmu }}}_{{it}}$ is a vector of departmental weather means computed as 20-year moving averages, ${{\gamma }_{j}t+\delta }_{j}{t}^{2}$ is a quadratic regional time trend accounting for agronomic improvements linked to orchard density, cultivars grown and crop load management, *ε*_*it*_ is a department and year-specific random error term and ${\alpha }_{i}$ is a departmental fixed effect capturing time-invariant unobservable characteristics that influence yields, such as soil type. The square indices attached to the vectors **x**_*it*_ and **x**_*it*_ − **μ**_*it*_ denote a Hadamard square power.

The weather variables we included in the model—that is, in ${{\bf{x}}}_{{it}}$—are average daily mean temperature and cumulated precipitation in each one of the four phenological phases (eight variables in total) as well as cumulated frost degree days during the blooming period, which is the equivalent of growing degree days but for temperatures below 0 °C. We computed frost degree days by first fitting an intradaily curve between minimum and maximum daily temperatures using the chillR package in R (ref. ^30^) and then summing hourly temperatures located below zero over the whole blooming period. We constructed all weather variables by averaging 25 × 25 km cell values from the EU Joint Research Centre’s AGRI4CAST Toolbox at the department level using weights reflecting the share of departmental apple surfaces falling within each cell, which we obtained from the French Ministry of Agriculture’s ‘Inventaire des vergers 2013’ database. Given the lack of precise phenology data available for the different growing areas included in our sample, we defined each phenological phase using fixed dates across locations as follows: 1 April until 15 May 15 for the blooming phase, 16 May until 30 June for the early growth/cell division phase, 1 July until 31 August for the late growth/cell expansion phase and 1 September until 31 October for the harvest phase. For the blooming phase, we made sure that the range of dates chosen encompasses all possible blooming dates of apples trees across France according to data from INRAE’s Phenoclim-Agroclim database. For the remaining phases, we based our decision on common acceptance in the ecophysiological literature that the cell division phase lasts between 35 and 45 days after full bloom, followed by the cell expansion phase, which extends until maturity^21^. Regarding the variables chosen to account for temperature, we tried alternative versions of our model using cumulated GDD5 and average maximum daily temperatures over each phase. While average maximum daily temperatures performed worse according to measures of fit, cumulative GDD5 performed comparably to average daily mean temperatures; however, we chose the latter for simplicity of interpretation and communication. We also tested augmented versions of our regression in which we added variables known to potentially have an effect on yields, such as chill units and weather conditions in the preharvest year. However, as these all led to similar results without raising the model’s explanatory power according to the leave-one-out *R*-squared, we stuck to our original specification to avoid overfitting the data (Supplementary Results section 1).

We estimated equation (1) using standard ordinary least squares, and clustered standard errors at the region–year level to account for potential intra-annual autocorrelation of error terms between departments belonging to the same region, as was done by Mérel and Gammans^8^ for the yields of annual crops. For local climate (${{\bf{\upmu }}}_{{it}}$), we used moving averages computed over the last 20 years before every observation except for the 10 years spanning from 1969 to 1978, for which we assumed a constant climate equal to the climate of the 1979–1988 decade given the unavailability of weather records for all years before 1979. Our use of moving averages instead of time-invariant averages like in the standard version of the model is motivated by two aspects. First, relying on fixed climatic means while temperature variables in most locations are non-stationary over the sample period can cause estimates of the ${\beta }_{3}$ coefficients to be biased. Second, from the perspective of farmers’ behaviour, it is conceptually more consistent to base climate—that is, weather expectations—solely on past observations, as in the study by Wimmer et al.^31^, rather than on an unequal mix of past and future observations, which would depend on the year considered in the sample.

As can be seen in equation (1), each of the weather variables (9 in total) enters the equation 3 times (for a total of 27 weather-related covariates): first through a linear term (${{\bf{x}}}_{{it}}$), second through a quadratic term (${{\bf{x}}}_{{it}}^{2}$) and finally through a so-called climate penalty term (${\left({{\bf{x}}}_{{it}}-{{\bf{\upmu }}}_{{it}}\right)}^{2}$), which accounts for yearly deviations around the variable’s mean. The only exception to this rule is the late frost variable, for which only a penalty term conditional on its deviation from the mean being positive—that is, the difference ${{{x}}}_{{it}}-{{{\mu }}}_{{it}}$ taking on a positive value—enters the equation. This is because, unlike temperature and precipitation, there is no rationale for allowing frost events that are less severe than expected to impose a yield penalty in the short run compared with the long run.

The climate penalty term ${{\mathbf{\upbeta}}_{\mathbf{3}}\,\left({{\bf{x}}}_{{it}}-{{\mathbf{\upmu }}}_{{it}}\right)}^{2}$ found in equation (1) is meant to capture the effect of climate change adaptation on apple yields in the long run. Its inclusion in the model supposes that, conditional on a given yearly weather realization, a location with a climate—that is, with a mean weather value—that is further away from the realization will fare worse than a location with a climate that is closer to it, provided that the coefficient ${\beta }_{3}$ takes on a negative value. As can be seen in Fig. 1b,f, this does not mean that any deviation of contemporary weather from the local mean (that is, any weather shock) will induce a yield loss relative to yields expected from the local climate, but rather that the effect of the deviation will be more negative than if the same deviation had occurred in the form of a long-term shift in climate, regardless of the sign of the effect (that is, positive or negative). In this respect, our penalty term differs from that of Moore and Lobell^9^, where the production function is quadratic in mean weather rather than in contemporary weather, an approach we avoid for the same reasons as those previously mentioned in the literature^8^. Instead, in our approach, equation (1) provides an alternative framework to distinguish between the short-run response (that is, the yield response to a weather shock) and the long-run response (that is, the yield response to a change in climate that stretches over many years and during which all production inputs, technologies and management practices can be adjusted). In this equation, the regression coefficients tied to the climate penalty term (${\mathbf{\upbeta}}_{\mathbf{3}}$) provide a simple way to test whether adaptation to climate has taken place in the past, as the climate penalty represents the difference between the expected (*E*) long-run response to climate (where, by definition, ${{\bf{x}}}_{{it}}={{\mathbf{\upmu }}}_{{it}}$) captured by equation (2) and the expected short-run response to weather (where the values of ${{\bf{x}}}_{{it}}$ and ${{\mathbf{\upmu }}}_{{it}}$ are allowed to differ) captured by equation (3). For each weather variable, the coefficient ${\beta }_{3}$ must be statistically significant and negative to conclude the existence of climate adaptation, as only in this case is the long-run response greater than its short-run equivalent for all changes in weather with respect to the prevailing local climate.

2 $$E\left(\,{y}_{\mathrm{LR}\,{it}}|{{\bf{x}}}_{{it}},t,{\alpha }_{i}\right)={\mathbf{\upbeta}}_{\mathbf{1}}{{\bf{x}}}_{{it}}+{\mathbf{\upbeta}}_{\mathbf{2}}{{\bf{x}}}_{{it}}^{2}+{{\gamma }_{j}t+\delta }_{j}{t}^{2}+{\alpha }_{i}$$

3 $$E\left({y}_{{\mathrm{SR}}\,{it}}|{{\bf{x}}}_{{it}},{{\mathbf{\upmu }}}_{{it}},t,{\alpha }_{i}\right)={\mathbf{\upbeta}}_{\mathbf{1}}{{\bf{x}}}_{{it}}+{\mathbf{\upbeta}}_{\mathbf{2}}{{\bf{x}}}_{{it}}^{2}+{\mathbf{\upbeta }}_{\mathbf{3}}{\left({{\bf{x}}}_{{it}}-{{\mathbf{\upmu }}}_{{it}}\right)}^{2}+{{\gamma }_{j}t+\delta }_{j}{t}^{2}+{\alpha }_{i}.$$

Details on how the long-run and short-run yield responses to temperature in different phenological phases (Fig. 1) were derived from equations (2) and (3) can be found in Supplementary Methods section 1.

### Defining harnessing and mitigating adaptation

For every temperature variable in our model for which the climate penalty coefficient (${\beta }_{3}$) is found to be negative and statistically significant at the 5% level, harnessing adaptation is defined as

4 $${A}_{\mathrm{harnessing}}={-\beta }_{3}{\left(T-\overline{T}\right)}^{2}\,\mathrm{when}\,\overline{T} < {T}_{\mathrm{LR}}^{* }\,\& \,T > \overline{T},$$

where T is the observed temperature, $\bar{T}$ is the local mean temperature calculated as a 20-year moving average on past observations and ${T}_{\mathrm{LR}}^{* }$ is the optimal growing temperature maximizing the long-run yield function $({T}_{\mathrm{LR}}^{* }=\frac{-{\beta }_{1}}{2{\beta }_{2}})$. Similarly, mitigating adaptation is defined as

5 $${A}_{\mathrm{mitigating}}={-\beta }_{3}{\left(T-\overline{T}\right)}^{2}\,\mathrm{when}\,(\overline{T} > {T}_{\mathrm{LR}}^{* }\,{or}\,{T}_{\mathrm{LR}}^{* }=\nexists )\,\& \,T > \overline{T},$$

where ${T}_{\mathrm{LR}}^{* }=\nexists$ means for the purpose of our analysis that ${T}_{\mathrm{LR}}^{* }$ is not statistically significant at the 5% level. In this case, mitigating adaptation not only reduces but completely eliminates any yield loss caused by the short-term mismatch between T and $\bar{T}$. Technical details on how adaptation expressed in percentage change with respect to the base year (1989) was decomposed into its harnessing and mitigating component, as shown in Fig. 2b, can be found in Supplementary Methods section 3.

### Yield responses to climate trends with and without adaptation

In the quasi-static context of a 1-year change in weather only, taking the difference between equations (2) and (3) gives the yield gap between the short-run and long-run yield response. However, this is not the case in the multiyear dynamic context of our study, where local climate is continuously updated through the rolling means in ${{\mathbf{\upmu}}}_{{it}}$. As a result, fitting equation (3) to climate trends produces a time series of long-run yield responses with respect to the climate prevailing in the starting year (either 1989 or 2020) rather than short-run yield responses, which would require the local climate to remain the same throughout the years. This is why our time series of yield responses to climate trends with adaptation (Fig. 2a,b,d,f) are derived from predicted yields given by equation (3) rather than equation (2), minus the quadratic time trends that account for time-variant factors not related to climate:

6 $${\hat{y}}_{it\,{\mathrm{Adaptation}}}={\alpha }_{i}+{\hat{\mathbf{\upbeta}}}_{\mathbf{1}}{{\bf{x}}}_{it}+{\hat{\mathbf{\upbeta}}}_{\mathbf{2}}{{\bf{x}}}_{it}^{\mathbf{2}}+{\hat{\mathbf{\upbeta}}}_{\mathbf{3}}{({{\bf{x}}}_{it}-{{\mathbf{\upmu}}}_{it})}^{2},$$

where ${\hat{y}}_{{it}}$ is the log of predicted yields. Following the logic stated further above, to obtain the time series of yield responses to climate trends without adaptation (Fig. 2a,e), we replaced the vector of climate means ${{\bf{\upmu }}}_{{it}}$ with the vector ${{\bf{\upmu }}}_{i0}$, the values of which stay constant through time:

7 $${\hat{y}}_{it\,{\mathrm{No}}\,{\mathrm{adaptation}}}={\alpha }_{i}+\,{\hat{\mathbf{\upbeta}}}_{\mathbf{1}}{{\bf{x}}}_{it}+{\hat{\mathbf{\upbeta}}}_{\mathbf{2}}{{\bf{x}}}_{it}^{2}+{\hat{\mathbf{\upbeta}}}_{\mathbf{3}}{({{\bf{x}}}_{it}-{{\mathbf{\upmu}}}_{i0})}^{2},$$

where ${{\bf{\upmu }}}_{i0}$ contains local climate values for the year where climate adaptation is assumed to stop taking place, which in our case is either the beginning of the sample period (1989) or its end (2020) when working with climate scenario data. It is important to note that for every weather variable the expression ${\left({{{x}}}_{{it}}-{{{\mu }}}_{{it}}\right)}^{2}\, < \,{\left({{{x}}}_{{it}}-{{{\mu }}}_{i0}\right)}^{2}$ holds true whenever ${{{x}}}_{{it}}$ is a monotonic function of time, which means that predicted yields with adaptation are higher than those without adaptation in any trending climate. Supplementary Methods section 9 shows both algebraically and graphically how taking the difference between equations (6) and (7) is equivalent to taking the difference between equations (2) and (3), with the additional advantage of taking into account the effect of weather shocks, to which growing systems can never be perfectly adapted. Technical details on how the yield responses displayed in Fig. 2 were transformed into percentage change with respect to the starting year (1989 or 2020), as well as information on how we estimated both the historical and future climate trends to which equations (6) and (7) were fitted, can be found in Supplementary Methods sections 2 and 7, respectively.

### Reporting summary

Further information on research design is available in the Nature Portfolio Reporting Summary linked to this article.

## Supplementary information

Supplementary Information Supplementary Tables 1–8, Figs. 1–3, Results and Methods.

Reporting Summary

Supplementary Data 1 Source data for all supplementary figures and tables except Supplementary Table 8.

## Source data

Source Data Fig. 1 Statistical source data for each graph in Fig. 1.

Source Data Fig. 2 Statistical source data for each graph in Fig. 2.

## Data Availability

All datasets used and generated as part of the research conducted in this Brief Communication are available via figshare at https://doi.org/10.6084/m9.figshare.c.8297458 (ref. ^32^). Publicly accessible raw input data used in this study can be found under the following links: (1) for time series of apple yields and apple area grown at the department level: https://agreste.agriculture.gouv.fr/agreste-web/disaron/SAANR_FRUITS/detail/ (only the times series for the 2000–2020 period are publicly available, the remaining chunk having been obtained directly from the French Ministry of Agriculture upon request); (2) for data on municipal-level orchard area across France: https://agreste.agriculture.gouv.fr/agreste-web/disaron/ChdAgr227/detail/; (3) for historical weather data: https://agri4cast.jrc.ec.europa.eu/dataportal; (4) for climate scenario data: https://www.drias-climat.fr/commande. Source data are provided with this paper.

## Code availability

All custom software code generated and used as part of this study—alongside all related datasets—is available via figshare at https://doi.org/10.6084/m9.figshare.c.8297458 (ref. ^32^).
