# Supplementary material for: Harnessing adaptation has increased French apple yields but heat stress mitigation will become essential by 2050
Source: Nat Food. 2026 Mar 31;7(4):321–7. doi: 10.1038/s43016-026-01333-0 (PMC13658012; doi:10.1038/s43016-026-01333-0)
Supplement: Supplementary file 1 [file 43016_2026_1333_MOESM1_ESM.pdf]

nature food

Supplementary information

https://doi.org/10.1038/s43016-026-01333-0

Harnessing adaptation has increased French apple yields but heat stress mitigation will become essential by 2050

In the format provided by the authors and unedited

Supplementary Results

1. Regression results for yield model

Table S1 displays the regression coefficients for four versions of our model that we estimated for robustness purposes without making changes to the weather-related variables it contains. Version 1 (in bold characters) is the main specification from which we obtained the coefficients used in our analysis. Version 2 contains quadratic time trends at the departmental level rather than at the regional level. Version 3 has yields not adjusted for biennial bearing as a dependent variable. Finally, Version 4 only considers deviations from the mean that are positive within its climate penalty terms in order to verify if the adaptation detected by the model really represents adaptation to rising temperatures over time. The coefficients are only shown for temperature and late frost variables, since taken together, precipitation variables have a very negligible impact on yields (Table S3). The abbreviation "PT" stands for the climate penalty terms which determine the presence of climate adaptation. It can be seen that while the magnitude and the level of significance of some effects slightly changes from one version to the other, the direction never does, thus confirming the robustness of our main specification.

Table S2 displays the regression coefficients of three other versions of our model that we estimated for robustness purposes (in addition to Version 1 in bold characters, which still represents our main specification), but this time making changes to the weather-related variables included. In Version 2, we added chill portions (based on the Dynamic Model for dormancy breaking) estimated using the chillR package in R to account for the effect of winter chill on yields, which turns out to be insignificant and does not add any explanatory power to the model according to the cross-validated leave-one-out R-squared ($LOOR^2$). This is not surprising given that winter chill is a requirement to meet rather than a climate factor with a continuous effect on apple yields and that minimum chill requirements have up until now been met almost each year in the vast majority of French apple-growing areas despite the warming trends. In Version 3, we added both cell division and cell expansion temperatures as well as precipitation in the previous year (t-1), since climatic conditions in the summer preceding flowering are known to potentially impact yields in the following year through bud initiation and development. While the results seem to show some impact of temperatures in the previous year, from a statistical perspective disentangling the effect between the current and previous year is a delicate task in a context where temperatures follow a clear upward trend over time, as this leads to collinearity between the regressors. Finally, in Version 4, we simply removed all precipitation-related variables from the regression. While this does not impact the direction of the temperature effects estimated from our main version, it changes their magnitude and causes a noticeable drop in explanatory power both according to the standard (R^2) and cross-validated leave-one-out R-squared ($LOOR^2$), thus dismissing any concern about overfitting when including precipitation variables in the regression. Moreover, since precipitation effects are simultaneous to the temperature effects, in that they are tied to the same phenological phases, excluding them could potentially bias the value of the temperature effects we rely on for our analysis, something we wish to avoid. As a final remark, the fact that the AIC and BIC never jointly conclude that any of the three additional versions tested is preferrable to Version 1 also points to the validity of our original specification.

Table S3 shows the regression coefficients tied to the precipitation variables for the four versions of our model shown in Table S1. As can be seen in the table, there is no single physiological phase for which precipitation has a significant concave effect on yields.

	**(1)**<br>**log(yield)**	(2)<br>log(yield)	(3)<br>log(yield)	(4)<br>log(yield)
Frost degree days	**-0.248*****<br>**(0.0666)**	-0.251***<br>(0.0675)	-0.277***<br>(0.0708)	-0.239***<br>(0.0675)
Frost degree days^2	**0.0827**<br>**(0.0539)**	0.0729<br>(0.0544)	0.0952<br>(0.0602)	0.0821<br>(0.0565)
PT frost degree days	**-0.000421**<br>**(0.0678)**	0.0180<br>(0.0700)	-0.00637<br>(0.0773)	-0.00441<br>(0.0708)
Blooming Tavg	**0.270****<br>**(0.0971)**	0.259**<br>(0.0960)	0.273*<br>(0.107)	0.358***<br>(0.105)
Early growth Tavg	**0.263****<br>**(0.0932)**	0.253*<br>(0.101)	0.239*<br>(0.105)	0.165*<br>(0.0836)
Late growth Tavg	**0.0474**<br>**(0.0902)**	0.123<br>(0.0960)	0.0735<br>(0.0973)	0.0863<br>(0.0896)
Harvest Tavg	**-0.0237**<br>**(0.0724)**	-0.0154<br>(0.0709)	-0.0197<br>(0.0779)	-0.0679<br>(0.0698)
Blooming Tavg^2	**-0.00806***<br>**(0.00359)**	-0.00753*<br>(0.00352)	-0.00796*<br>(0.00394)	-0.0113**<br>(0.00379)
Early growth Tavg^2	**-0.00781****<br>**(0.00252)**	-0.00746**<br>(0.00272)	-0.00727*<br>(0.00284)	-0.00496*<br>(0.00230)
Late growth Tavg^2	**-0.000747**<br>**(0.00207)**	-0.00250<br>(0.00221)	-0.00116<br>(0.00224)	-0.00133<br>(0.00208)
Harvest Tavg^2	**0.000126**<br>**(0.00236)**	-0.000133<br>(0.00229)	-0.0000875<br>(0.00255)	0.00134<br>(0.00231)
PT blooming Tavg	**-0.0260*****<br>**(0.00683)**	-0.0265***<br>(0.00753)	-0.0295***<br>(0.00764)	-0.0239**<br>(0.00727)
PT early growth Tavg	**0.00512**<br>**(0.00483)**	0.00489<br>(0.00493)	0.00722<br>(0.00532)	-0.000751<br>(0.00965)
PT late growth Tavg	**-0.0144***<br>**(0.00624)**	-0.0135*<br>(0.00659)	-0.0182**<br>(0.00691)	-0.0185*<br>(0.00926)
PT harvest Tavg	**0.00849**<br>**(0.00453)**	0.00770<br>(0.00463)	0.0107*<br>(0.00478)	0.00811<br>(0.00657)
Quad. time trends	**Region**	Department	Region	Region
Department FE	**Yes**	Yes	Yes	Yes
SE cluster	**Region-Year**	Region-Year	Region-Year	Region-Year
AR(1) first stage	**Yes**	Yes	No	Yes
PT deviations	**All**	All	All	Positive only
Observations	**1024**	1024	1024	1024
R2	**0.659**	0.716	0.631	0.655
LOOR2	**0.596**	0.623	0.561	0.590

Standard errors in parentheses

* p<0.05, ** p<0.01, *** p<0.001

Table S1: Temperature-related estimates from the main regression and alternate versions containing the same weather variables. All coefficients are estimated using standard OLS and their associated standard errors are two-way clustered at the region-year level. P-values(*) are derived from two-sided t-tests.

	**(1)**<br>**log(yield)**	(2)<br>log(yield)	(3)<br>log(yield)	(4)<br>log(yield)
Frost degree days	**-0.248*** **	-0.251***	-0.277***	-0.239***
Frost degree days^2	**0.0827**	0.0729	0.0952	0.0821
PT frost degree days	**-0.000421**	0.0180	-0.00637	-0.00441
Blooming Tavg	**0.270** **	0.259**	0.273*	0.358***
Early growth Tavg	**0.263** **	0.253*	0.239*	0.165*
Late growth Tavg	**0.0474**	0.123	0.0735	0.0863
Harvest Tavg	**-0.0237**	-0.0154	-0.0197	-0.0679
Blooming Tavg^2	**-0.00806***	-0.00753*	-0.00796*	-0.0113**
Early growth Tavg^2	**-0.00781** **	-0.00746**	-0.00727*	-0.00496*
Late growth Tavg^2	**-0.000747**	-0.00250	-0.00116	-0.00133
Harvest Tavg^2	**0.000126**	-0.000133	-0.0000875	0.00134
PT blooming Tavg	**-0.0260*** **	-0.0265***	-0.0295***	-0.0239**
PT early growth Tavg	**0.00512**	0.00489	0.00722	-0.000751
PT late growth Tavg	**-0.0144***	-0.0135*	-0.0182**	-0.0185*
PT harvest Tavg	**0.00849**	0.00770	0.0107*	0.00811
Chill portions		-0.104		
Chill portions^2		0.000627		
PT chill portions		-0.00122		
Early growth Tavg(t-1)			0.152	
Late growth Tavg(t-1)			-0.250*	
Early growth Tavg(t-1)^2			-0.00382	
Late growth Tavg(t-1)^2			0.00635*	
PT early growth Tavg(t-1)			-0.00275	
PT late growth Tavg(t-1)			-0.00665	
Early growth prec(t-1)			0.00171*	
Late growth prec(t-1)			0.000153	
Early growth prec(t-1)^2			-0.00000864	
Late growth prec(t-1)^2			-0.00000315	
PT early growth prec(t-1)			0.00000513	
PT late growth prec(t-1)			0.00000558	
Quad. time trends	**Region**	Department	Region	Region
Department FE	**Yes**	Yes	Yes	Yes
SE cluster	**Region-Year**	Region-Year	Region-Year	Region-Year
AR(1) first stage	**Yes**	Yes	Yes	Yes
PT deviations	**All**	All	All	All
Precipitation variables	**Yes**	Yes	Yes	No
Observations	**1024**	1024	1024	1024
R2	**0.659**	0.661	0.668	0.642
LOOR2	**0.596**	0.596	0.593	0.585
AIC	**-340**	-339	-352	-313
BIC	**50**	65	94	18

* p<0.05, ** p<0.01, *** p<0.001

Table S2: Estimates from the main regression and alternate versions containing different weather variables. All coefficients are estimated using standard OLS and their associated standard errors are two-way clustered at the region-year level. Standard errors are not shown to limit the size of the table but can be found in the source data file. P-values(*) are derived from two-sided t-tests.

	**(1)**<br>**log(yield)**	(2)<br>log(yield)	(3)<br>log(yield)	(4)<br>log(yield)
Blooming prec	**-0.000646**	-0.000739	-0.000966	-0.000494
	**(0.000986)**	(0.000985)	(0.00104)	(0.000951)
Early growth prec	**-0.00120**	-0.00107	-0.00112	-0.00103
	**(0.000776)**	(0.000815)	(0.000875)	(0.000754)
Late growth prec	**-0.000779**	-0.000627	-0.000586	-0.000664
	**(0.000533)**	(0.000522)	(0.000545)	(0.000529)
Harvest prec	**-0.000805**	-0.000648	-0.000886	-0.000919
	**(0.000682)**	(0.000712)	(0.000739)	(0.000685)
Blooming prec^2	**-0.00000129**	-0.000000583	-0.000000363	-0.00000178
	**(0.00000531)**	(0.00000529)	(0.00000558)	(0.00000507)
Early growth prec^2	**0.00000829**	0.00000806	0.00000837	0.00000703
	**(0.00000478)**	(0.00000511)	(0.00000536)	(0.00000451)
Late growth prec^2	**0.00000661***	0.00000649*	0.00000618*	0.00000620*
	**(0.00000297)**	(0.00000309)	(0.00000301)	(0.00000288)
Harvest prec^2	**0.00000199**	0.00000172	0.00000215	0.00000245
	**(0.00000230)**	(0.00000240)	(0.00000250)	(0.00000230)
PT blooming prec	**0.00000410**	0.00000262	0.00000473	0.00000423
	**(0.00000585)**	(0.00000585)	(0.00000616)	(0.00000555)
PT early growth prec	**-0.00000621**	-0.00000521	-0.00000737	-0.00000399
	**(0.00000569)**	(0.00000604)	(0.00000642)	(0.00000537)
PT late growth prec	**-0.00000289**	-0.00000386	-0.000000876	-0.00000303
	**(0.00000576)**	(0.00000613)	(0.00000571)	(0.00000569)
PT harvest prec	**0.000000201**	0.000000275	5.42e-08	-0.000000440
	**(0.00000220)**	(0.00000224)	(0.00000230)	(0.00000217)
Quad. time trends	**Region**	Department	Region	Region
Department FE	**Yes**	Yes	Yes	Yes
SE cluster	**Region-Year**	Region-Year	Region-Year	Region-Year
AR(1) first stage	**Yes**	Yes	No	Yes
PT deviations	**All**	All	All	Positive only
Observations	**1024**	1024	1024	1024
R2	**0.659**	0.716	0.631	0.655
LOOR2	**0.596**	0.623	0.561	0.590

Standard errors in parentheses

* p<0.05, ** p<0.01, *** p<0.001

Table S3: Precipitation-related estimates from the main regression and alternate versions containing the same weather variables. All coefficients are estimated using standard OLS and their associated standard errors are two-way clustered at the region-year level. P-values(*) are derived from two-sided t-tests.

2. Effect of climate trends on yields

Table S4 shows the combined effect of climate trends on apple yields at the department level for the sample period (1989-2020) expressed as a percentage change from 1989 yields. **Tables S5 & S6** contain the same information for the forecast period (2020-2050) under the RCP 4.5 and 8.5 emissions pathways respectively, the only difference being that percentage changes in yield are expressed relative to their 2020 value. **Figure S1** is the direct equivalent of **Figures 2e & 2f** in the Main Text, but for the RCP 8.5 emissions pathway. The slightly different results obtained for both pathways are due to cell expansion phase temperatures increasing more under the RCP 8.5 scenario, combined with the fact that growing systems can mitigate the impacts of heat stress in that phase as opposed to the cell division phase (see **Figure 1e** in the Main Text).

Department (INSEE number)	Full adaptation			No adaptation			Mitigation adaptation only		
	Δ% yield	Δ% yield min	Δ% yield max	Δ% yield	Δ% yield min	Δ% yield max	Δ% yield	Δ% yield min	Δ% yield max
4	7.7	0.5	14.9	-1.0	-7.6	5.6	0.2	-6.6	7.0
5	11.2	2.9	19.6	2.5	-5.2	10.2	3.6	-4.3	11.5
7	18.1	6.9	29.3	-0.6	-10.2	8.9	2.9	-7.0	12.7
11	-1.5	-7.4	4.5	-8.2	-13.8	-2.6	-6.2	-11.9	-0.5
13	-3.5	-8.0	1.0	-8.4	-12.8	-4.0	-7.5	-11.9	-3.0
16	15.1	5.5	24.7	2.8	-3.3	8.8	5.3	-1.7	12.3
18	13.0	5.0	21.1	2.0	-4.8	8.9	4.7	-2.7	12.2
19	15.3	6.0	24.6	4.9	-0.6	10.5	6.5	0.4	12.7
23	10.8	2.2	19.5	-0.2	-7.1	6.8	1.5	-5.9	8.9
24	11.1	3.6	18.6	3.1	-1.7	7.8	4.2	-1.0	9.4
26	6.2	-1.2	13.6	-8.2	-14.4	-2.0	-6.3	-12.6	-0.1
30	-5.6	-12.0	0.7	-14.0	-20.5	-7.6	-11.9	-18.3	-5.6
33	5.2	-0.4	10.7	-0.3	-4.3	3.7	0.6	-3.7	4.9
34	-6.3	-13.8	1.2	-16.4	-23.9	-8.9	-13.1	-20.5	-5.8
37	18.7	7.6	29.7	4.9	-3.3	13.2	8.9	-0.5	18.4
38	11.2	2.2	20.2	-7.0	-14.7	0.7	-4.2	-11.9	3.5
40	20.4	7.9	32.8	6.4	-0.6	13.4	9.4	1.4	17.3
42	15.9	5.7	26.2	-4.6	-13.3	4.2	-1.0	-9.8	7.8
47	12.5	3.2	21.8	-0.9	-7.1	5.3	3.5	-3.5	10.6
49	12.8	4.5	21.1	2.4	-3.8	8.5	5.0	-2.0	12.0
64	19.0	5.9	32.1	5.5	-2.4	13.3	7.6	-0.9	16.0
66	21.7	9.1	34.4	9.2	-2.3	20.6	11.9	0.1	23.7
67	17.9	7.8	28.0	-1.3	-10.6	8.0	3.7	-5.6	13.1
69	16.0	4.4	27.6	-10.4	-22.0	1.3	-3.9	-15.2	7.4
72	17.6	7.3	27.8	4.2	-3.0	11.4	7.7	-0.6	16.0
73	33.2	12.1	54.2	10.0	-3.8	23.7	14.9	0.3	29.4
74	15.6	-21.7	52.9	-3.8	-31.1	23.5	-0.6	-29.2	28.0
79	21.1	9.8	32.4	8.9	0.2	17.6	12.6	2.8	22.4
82	10.8	2.6	19.1	-1.0	-6.6	4.5	2.4	-3.9	8.7
84	-4.1	-9.2	1.0	-9.9	-14.8	-5.0	-8.6	-13.5	-3.6
86	36.1	19.9	52.4	16.5	2.0	31.1	22.3	6.6	38.1
87	14.8	5.1	24.6	4.5	-1.9	11.0	5.9	-1.0	12.9

Table S4: Combined effect of climate trends on departmental apple yields over the historical period (1989-2020). All yield changes are expressed in percentage points from the base year (1989). Values in the case of full or zero adaptation were computed by fitting the estimates from Table S1's main version to Equation 10 found in the Supplementary Methods section, while Equation 14 was used in the case of mitigation adaptation only taking place. Minimum and maximum yield changes were computed according to a 95% prediction interval obtained from the delta method.

Department	Full adaptation			No adaptation			Mitigation adaptation only		
(INSEE number)	Δ% yield	Δ% yield min	Δ% yield max	Δ% yield	Δ% yield min	Δ% yield max	Δ% yield	Δ% yield min	Δ% yield max
4	9.5	3.6	15.3	7.0	1.9	12.1	7.7	2.4	13.0
5	19.9	10.7	29.1	17.9	2.5	33.3	18.5	3.0	34.0
7	0.5	-3.7	4.7	-1.8	-5.9	2.2	-1.2	-5.3	3.0
11	-7.0	-11.8	-2.3	-7.4	-11.3	-3.5	-6.9	-10.8	-2.9
13	-4.6	-9.5	0.3	-5.4	-9.9	-0.8	-5.0	-9.6	-0.4
16	1.8	-2.8	6.3	0.4	-3.9	4.7	0.4	-3.8	4.7
18	1.4	-2.4	5.3	0.5	-3.7	4.7	0.5	-3.7	4.8
19	5.6	-0.2	11.4	4.6	-0.3	9.4	4.7	-0.2	9.6
23	10.5	5.4	15.5	9.9	5.0	14.7	9.9	5.0	14.7
24	2.4	-2.2	7.0	1.6	-2.5	5.7	1.7	-2.4	5.7
26	1.0	-2.8	4.7	-0.8	-4.4	2.7	-0.6	-4.2	3.1
30	-4.9	-9.9	0.2	-6.1	-10.6	-1.6	-5.6	-10.2	-1.1
33	-0.4	-4.9	4.2	-0.7	-4.8	3.4	-0.6	-4.7	3.5
34	-5.3	-9.5	-1.0	-6.1	-10.0	-2.2	-5.7	-9.6	-1.8
37	1.4	-2.3	5.0	-0.2	-4.1	3.8	-0.1	-4.1	3.9
38	-1.3	-4.9	2.3	-3.0	-6.9	0.9	-2.7	-6.6	1.3
40	0.5	-4.7	5.7	-1.3	-5.7	3.1	-1.1	-5.5	3.4
42	5.0	0.0	10.0	2.8	-1.6	7.2	3.2	-1.3	7.8
47	-3.4	-7.6	0.9	-4.3	-8.4	-0.2	-3.8	-7.9	0.3
49	3.2	-0.5	6.9	2.0	-1.7	5.6	2.0	-1.6	5.6
64	1.3	-4.3	7.0	-1.4	-5.5	2.7	-1.3	-5.4	2.8
66	-6.9	-13.7	-0.2	-6.9	-11.6	-2.1	-5.7	-10.5	-0.9
67	4.7	-0.2	9.6	3.7	-0.9	8.2	3.8	-0.8	8.4
69	-0.7	-5.1	3.7	-2.9	-6.9	1.1	-2.4	-6.5	1.6
72	2.8	-0.9	6.5	1.5	-2.2	5.3	1.5	-2.2	5.3
73	14.2	3.7	24.8	10.0	0.9	19.2	10.7	1.4	20.0
74	12.5	4.3	20.6	9.8	2.3	17.3	10.0	2.5	17.5
79	3.8	-0.7	8.3	1.9	-2.6	6.4	2.0	-2.5	6.5
82	-3.8	-7.7	0.1	-5.5	-9.1	-1.9	-5.2	-8.8	-1.5
84	-4.7	-9.7	0.2	-5.5	-10.1	-1.0	-5.2	-9.9	-0.6
86	1.7	-2.9	6.2	0.0	-4.6	4.6	0.2	-4.4	4.8
87	8.7	2.8	14.6	7.9	2.7	13.1	7.9	2.7	13.1

Table S5: Combined effect of climate trends on departmental apple yields in the RCP 4.5 scenario (2020-2050). All yield changes are expressed in percentage points from the base year (2020). Values in the case of full or zero adaptation were computed by fitting the estimates from Table S1's main version to Equation 10 found in the Supplementary Methods section, while Equation 14 was used in the case of mitigation adaptation only taking place. Minimum and maximum yield changes were computed according to a 95% prediction interval obtained from the delta method.

Department	Full adaptation			No adaptation			Mitigation adaptation only		
(INSEE number)	Δ% yield	Δ% yield min	Δ% yield max	Δ% yield	Δ% yield min	Δ% yield max	Δ% yield	Δ% yield min	Δ% yield max
4	4.9	-0.8	10.7	-1.5	-5.8	2.7	3.2	-1.6	7.9
5	13.1	7.1	19.1	5.5	0.5	10.6	11.5	6.1	17.0
7	4.1	-2.9	11.1	-1.4	-6.2	3.5	3.1	-2.2	8.3
11	0.0	-4.9	4.8	-4.6	-8.2	-0.9	0.1	-4.2	4.4
13	-2.8	-7.8	2.3	-4.9	-9.3	-0.5	-2.9	-7.5	1.7
16	4.3	0.5	8.2	0.2	-2.1	2.6	4.0	0.7	7.3
18	5.1	1.2	9.0	1.4	-1.1	3.9	5.5	2.1	8.8
19	2.7	-0.5	5.9	-0.4	-2.8	2.0	3.2	0.2	6.3
23	4.9	1.5	8.3	1.8	-0.8	4.3	5.3	2.0	8.5
24	1.9	-0.9	4.7	-1.1	-3.1	1.0	2.3	-0.4	5.0
26	1.8	-3.3	6.8	-2.7	-6.4	1.0	1.2	-2.8	5.2
30	-4.4	-9.5	0.8	-6.8	-11.3	-2.3	-4.2	-9.0	0.5
33	1.0	-2.2	4.2	-2.3	-4.6	-0.1	1.4	-1.7	4.6
34	-5.1	-10.3	0.1	-7.1	-11.7	-2.5	-4.6	-9.4	0.1
37	4.3	1.1	7.5	1.6	-0.6	3.8	4.9	1.9	7.9
38	0.5	-4.5	5.4	-4.3	-7.9	-0.6	-0.2	-3.9	3.6
40	0.9	-4.3	6.1	-5.6	-9.4	-1.7	1.6	-3.4	6.7
42	0.7	-3.3	4.6	-3.3	-6.4	-0.2	0.6	-2.6	3.8
47	-1.7	-5.3	2.0	-5.9	-8.6	-3.2	-1.0	-4.4	2.4
49	3.1	0.8	5.4	1.4	-0.4	3.2	3.5	1.2	5.8
64	1.3	-3.9	6.6	-5.1	-9.2	-1.0	2.0	-3.3	7.3
66	2.6	-2.9	8.1	-5.5	-9.3	-1.7	2.7	-1.9	7.4
67	-2.9	-5.7	-0.1	-5.5	-8.6	-2.4	-2.3	-5.3	0.7
69	-0.9	-5.4	3.6	-5.2	-8.9	-1.5	-0.7	-4.5	3.0
72	3.5	0.8	6.2	1.5	-0.5	3.5	4.0	1.5	6.6
73	2.7	-5.1	10.5	-3.3	-8.5	1.9	1.7	-3.7	7.0
74	5.0	-2.6	12.6	0.2	-4.6	5.1	4.4	-0.8	9.6
79	6.0	2.2	9.7	3.1	0.3	5.8	5.6	2.3	9.0
82	-0.3	-3.9	3.3	-3.7	-6.2	-1.1	0.1	-3.2	3.3
84	-3.3	-8.2	1.6	-5.9	-10.2	-1.6	-3.6	-8.1	1.0
86	5.4	1.4	9.4	0.8	-1.4	3.0	5.4	2.1	8.7
87	6.0	2.6	9.5	2.8	0.0	5.5	6.2	2.8	9.7

Table S6: Combined effect of climate trends on departmental apple yields in the RCP 8.5 scenario (2020-2050). All yield changes are expressed in percentage points from the base year (2020). Values in the case of full or zero adaptation were computed by fitting the estimates from Table S1's main version to Equation 10 found in the Supplementary Methods section, while Equation 14 was used in the case of mitigation adaptation only taking place. Minimum and maximum yield changes were computed according to a 95% prediction interval obtained from the delta method.

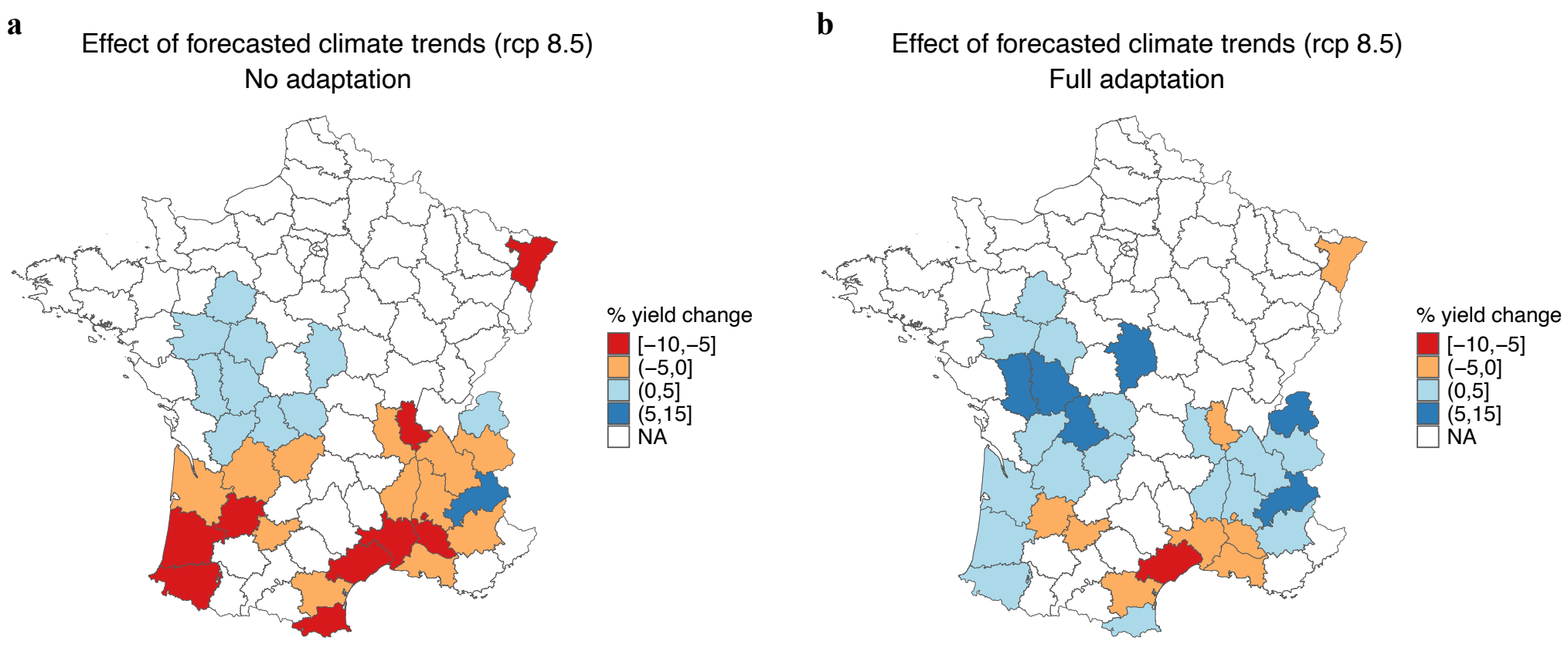

Figure S1: Maps of the combined effect of climate trends on departmental apple yields in the RCP 8.5 scenario (2020-2050). The values displayed correspond to mean yield changes shown in Table S6.

3. Regression results for geographic adaptation

Table S7 displays the results from the linear regression explained in section 6 of the Supplementary Methods. The low magnitude of the coefficient of interest (0.025% increase in the departmental share of the national apple area for every 1% increase in the effect of climate trends on yields) together with the absence of any statistical significance suggest that no geographic relocation has taken place as a way to adapt to climate change over the historical period.

	(1)<br>Δ%ha
Effect of climate trends<br>(%Δyield)	0.0252<br>(0.0281)
Predicted yield 2020<br>(quintals/ha)	-0.00315<br>(0.00367)
Constant	0.862<br>(1.641)
Observations	32
R2	0.067

Standard errors in parentheses

* p<0.05, ** p<0.01, *** p<0.001

Table S7: Estimates from the regression testing for geographic relocation as a climate change adaptation strategy. All coefficients are estimated using standard OLS and their associated standard errors are heteroskedasticity-robust. P-values(*) are derived from two-sided t-tests.

4. Descriptive data on varieties grown across France

Figure S2 displays the change in the relative importance of three main cultivars (Golden Delicious, Granny Smith and Gala) and of cultivars other than those three according to the area cultivated. The period shown corresponds to the sample period covered in the analysis and the statistics are computed using the French Ministry of Agriculture's data on fruit production surfaces (AGRESTE).

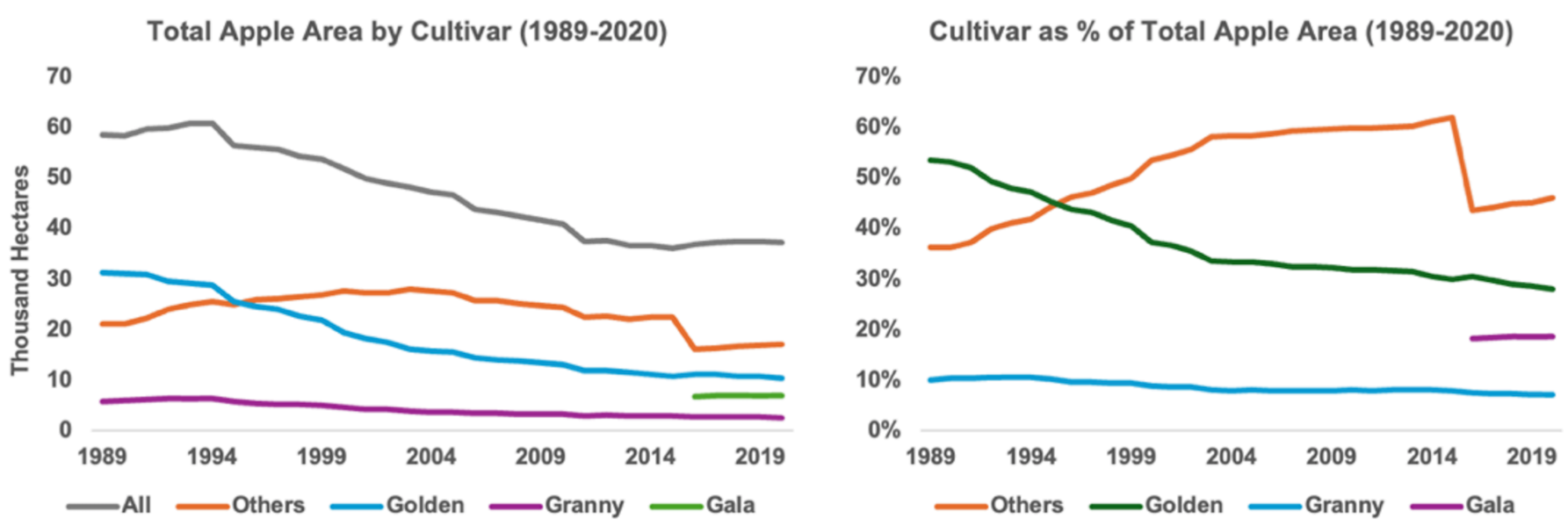

Figure S2: Changes in varietal composition of French apple production from 1989 until 2020. The data shown encompasses all apple-growing areas across France.

5. Comparison of empirical and biological optima

To ensure the physiological validity of the statistical model employed, **Table S8** compares empirical temperature optima derived from model estimates with known biological optima from the plant physiology literature. Long-run optima are unique for each phase, while short-run optima depend on the local mean and were hence calculated both at the minimum and maximum local mean observed at the end of the sample period. For comparative purposes, empirical values all correspond to mean daily maximum temperatures, which were obtained from fitting pooled simple linear regressions with the values of either x^*_{SR} or x^*_{LR}. More detail on the methods used can be found in Section 10 of the Supplementary Methods.

**Phenological phase**	**Empirical optima (mean daily Tmax, in °C)**		**Biological optimum (Tmax, in °C)** (Range of values obtained from the literature)
	Short-run – full range as of 2020 ($Tmax$ associated with: $x_{SR}^{*} = \frac{2\beta_3\mu - \beta_1}{2(\beta_2 + \beta_3)}$)	Long-run ($Tmax$ associated with: $x_{LR}^{*} = \frac{-\beta_1}{2\beta_2}$)	
Blooming phase	[17.6; 21.1]	22.0	[18.0; 25.0]
Early growth/cell division	21.8	21.8	[19.0; 25.0]
Late growth/cell expansion	[26.1; 31.3]	NA	[25.0; 33.0]

Table S8: Comparison of empirical and biological temperature optima for apple yields. Empirical temperature optima were obtained through fitting the estimates from Table S1's main version to Equation 30 (long-run) or Equation 31 (short-run) found in the Supplementary Methods section, while biological optima are derived from current knowledge in the eco physiological literature[21,24,25,26]. In the early growth phase, the empirical short-term optimal temperature is the same across all departments and is equal to the long-run optimum, given that adaptation is statistically no different from 0 in this phase (see Table S1). For the late growth phase, no empirical long-run optimum is given since only deviations from local means have a statistically significant impact on yields (See Table S1).

Supplementary Methods

1. Long run and short run yield responses to temperature changes

To obtain the long run yield responses displayed in **Figure 1** for each of the first two phenological phases**,** we use **Equation 2** in the Methods as a starting point and keep only the coefficients and variables associated with temperature in each desired phase, after which we transform the new equation so as to obtain a percentage change in yields:

$$\%\Delta\hat{y}_{LR} = 100 \times \left(e^{\Delta T(\hat{\beta}_1+\hat{\beta}_2(2T+\Delta T))} - 1\right) \ (8)$$

where ΔT and ΔT^2 represent the change in temperature and in the square value of temperature respectively when moving from $\bar{T}$ (the surface-weighted average national temperature in 2020) to $(\bar{T}+1)$ or $(\bar{T}+2)$. For the short run yield responses, we follow the same procedure, but use **Equation 3** in the Methods as a starting point:

$$\%\Delta\hat{y}_{SR} = 100 \times \left(e^{\Delta T(\hat{\beta}_1+\hat{\beta}_2(2T+\Delta T)+\hat{\beta}_3\Delta T)} - 1\right) \ (9)$$

where $(\Delta T)^2$ represents the square value of the deviation from the mean temperature $\bar{T}$ (either 1°C or 2°C).

2. Yield responses to climate trends expressed in percentage change

The relative changes in yield at the departmental level shown in **Figure 2** can be expressed through the following transformation of **Equations 6** & **7** in the Methods:

$$\%\Delta\hat{y}_{it_A} = 100 \times \left(e^{\left(\hat{y}_{it_A}-\hat{y}_{iT_{0A}}\right)} - 1\right) \ (10)$$

where A identifies whether the fitted yields include climate adaptation or not (i.e whether the value is taken from **Equation 6** or from **Equation 7**) and T_0 represents the reference year (either 1989 or 2020). For the purpose of computation, especially for the calculation of confidence intervals using the delta method, it proves useful to rely on an alternative condensed version of **Equation 10** expressed in matrix notation:

$$\%\Delta\hat{Y}_{it_A} = 100 \times \left(e^{\Delta X_{it_A}\hat{\beta}} - 1\right) \ (11)$$

where ΔX_{it_A} is the matrix containing the differences in the value of the weather variables found in **Equation 6** or **7** (depending on the value of A) for each department i between the year t and the reference year T_0 and where $\hat{\beta}$ represents the vector of estimated beta coefficients associated with these variables. To obtain the same values, but at the national level, such as those shown in **Figure 2a & 2b**, we use a weighted-average transformation of **Equation 11**:

$$\%\Delta\hat{Y}_{FRt_A} = 100 \times \left(e^{\left[\left({W_{it}}^T X_{it_A} - {W_{iT_0}}^T X_{iT_{0A}}\right)\hat{\beta}\right]} - 1\right) \ (12)$$

where X_{it_A} and $X_{iT_{0A}}$ represent the matrices containing the values of the weather variables found in **Equation 6** or **7** for each department i in year t and T_0 respectively and where W_{it} and W_{iT_0} represent the vectors containing the share of the national apple area tied to each department i in year t and T_0 respectively.

3. **Decomposing climate adaptation**

To obtain the effect of climate trends on departmental yields when only mitigating adaptation is at play (**Tables S4-S6**), the first step is to add the contribution of "mitigating adaptation" to predicted yields *without adaptation* (**Equation 7** in the Methods):

$$\hat{y}_{it_{mitigation}} = \hat{y}_{it_{No\ adaptation}} + \hat{B}_{3,LG}\left[\left(T_{it,LG} - \bar{T}_{it,LG}\right)^2 - \left(T_{it,LG} - \bar{T}_{iT_0,LG}\right)^2\right] (13)$$

where $\hat{B}_{3,LG}\left[\left(T_{it,LG} - \bar{T}_{it,LG}\right)^2 - \left(T_{it,LG} - \bar{T}_{iT_0,LG}\right)^2\right]$ denotes the difference between **Equations 6 & 7** in the Methods for temperature variables tied to the late growth/cell expansion phase, it being the only phase satisfying **Equation 5** in the Methods. This difference is always positive given that $\hat{B}_{3,LG}$ is negative. To obtain the exact values displayed in the third column of **Tables S4-S6,** we then apply **Equation 10** to the results of **Equation 13**:

$$\%\Delta\hat{y}_{it_{mitigation}} = 100 \times \left(e^{\left(\hat{y}_{it_{mitigation}} - \hat{y}_{iT_{0_{mitigation}}}\right)} - 1\right) (14)$$

Analogically, the national yield time series when only mitigating adaptation is at play is given by:

$$\%\Delta\hat{y}_{FRt_{mitigation}} = 100 \times \left(e^{\left(\sum_{i=1}^{32} w_{it}\hat{y}_{it_{mitigation}} - \sum_{i=1}^{32} w_{iT_0}\hat{y}_{iT_{0_{mitigation}}}\right)} - 1\right) (15)$$

where w_{it} and w_{iT_0} represent the share of national apple area tied to each department i in year t and T_0 respectively. To finally decompose total climate adaptation into harnessing and mitigating adaptation, as done in **Figure 2b**, we use the scalar results from **Equation 12** together with the result from **Equation 15**:

$$\hat{A}_{harnessing} = \%\Delta\hat{y}_{FRt_{adaptation}} - \%\Delta\hat{y}_{FRt_{mitigation}} (16)$$

$$\hat{A}_{mitigation} = \%\Delta\hat{y}_{FRt_{mitigation}} - \%\Delta\hat{y}_{FRt_{no\ adaptation}} (17)$$

4. **Estimating historical climate trends**

To estimate the climate trends whose predicted values for the year 2020 we fit to **Equations 6 & 7** in the Methods in order to understand the effect of past climate change on yields, we first linearly regress each climate variable included in our model over time. We do so separately for each department in order to estimate local climate trends, after which we compute predicted values using the coefficient estimate of time provided by each regression:

$$\hat{x}_{it} = \hat{\delta}_i + \hat{b}_i t \ (18)$$

where t represents the number of years that have passed since 1989 and $\hat{b}_i$ the estimated linear effect of time for each department. Finally, we recompute all the 20-year moving averages which enter the "climate penalty" terms of our model using the predicted climate values $\hat{x}_{it}$.

5. **Estimating future climate trends**

To estimate the climate trends whose predicted values for the year 2050 we fit to **Equations 6 & 7** in the Methods in order to understand the effect of future climate change on yields, we use climate data from the RCP 4.5 scenario, which is generally considered a compromise between best-case and worst-case warming prognoses. In the Supplementary Results, we also show the results we obtain when using data from the RCP 8.5 scenario. The data is drawn from the

CNRM-ALADIN regional climate model for France, made accessible under the French government's Projet DRIAS. Since the data provided has 8x8km resolution compared to 25x25km resolution for the historical climate data, we match the grid cells from both data sources based on the distance between the cells' central coordinates. Since this method has shortcomings in terms of accuracy, we do not merge the time series from both data sources together, but rather apply the effect of time observed in the scenario data to the historical data for out-of-sample prediction. We thus assume that the levels of both time series may not match over the forecast period, but that their trends should. As a first step, we estimate climate trends through linear regression over our sample period (1989-2020) as was done for historical data, but this time using the climate scenario data, whose reference period goes from 1951 to 2005 and forecast period from 2006 to 2100. Using the regression estimates, we then compute predicted climate values at the end of our sample period (2020), as shown in **Equation 18**. As a second step, we repeat the first step, the only difference being that we perform the regressions over the 1989-2050 period rather than the 1989-2020 period, so as to obtain predicted climate values at the end of our forecast period (2050). The last step consists in linearly interpolating the predicted values from both years (2020 and 2050) and using the resulting linear relationship to project out-of-sample climate trends:

$$\hat{x}_{it_A} = \hat{x}_{i2020_A} + \left(\frac{\hat{x}_{i2050_S} - \hat{x}_{i2020_S}}{30}\right) \times t \ (19)$$

where t represents the number of years that have passed since 2020 and where A and S stand for actual climate data (historical climate trends and their forecasted continuation into the future) and for scenario data (climate trends derived from RCP 4.5 or 8.5 data). By following this method, we project future climate trends which are coherent with the climate differences observed in the scenario data over our forecast period (2020-2050), conditional on expected climate values at the end of our sample period (2020) given historical time trends.

6. Testing for climate adaptation through geographic relocation

To test whether apple production has purposefully relocated to French departments whose apple yields have benefited from the effect of climate trends in the past, we estimate a simple linear regression in which we test the significance of the relationship between the change in each department's share of the national apple area from 1989 until 2020 and the effect of local climate trends on yields over the same period (**Equation 10**). In this regression, we control for predicted local yields in 2020 obtained from quadratic time trends also estimated through simple linear regression in order to disentangle relocation driven by climate change from relocation driven by other agronomic factors impacting yields. Thus, as a first step, we linearly regress local yields on a second order polynomial of time:

$$y_{it_{actual}} = \delta_i + B_{1i}t + B_{2i}t^2 + \varepsilon_{it} \ (20)$$

where $y_{it_{actual}}$ represents each department's actual yield in year t. Then, as a second step, we estimate the regression in which we test the effect of yield changes induced by climate trends on each department's share of the national apple area while controlling for predicted actual yields at the departmental level:

$$\Delta a_{i2020} = \gamma_i + B_{1i}\%\Delta\hat{y}_{i2020_{Adaptation}} + B_{2i}\hat{y}_{i2020_{actual}} + \varepsilon_i \ (21)$$

where Δa_{i2020} represents each department's change in its share of the national apple area from 1989 until 2020, $\%\Delta\hat{y}_{i2020_{Adaptation}}$ represents the outcome from **Equation 10** *with*

adaptation and $\hat{y}_{i2020_{actual}}$ stands for each department's predicted yield in 2020 obtained from **Equation 20**.

7. Climate-related effect of geographic relocation on national yields

To estimate the contribution to national yields of production relocation through climate, as shown in **Figure 2b**, we apply the logic of counterfactual analysis to predicted yields from climate by comparing the value of predicted national yields *with adaptation* when the spatial distribution of production changes over time to the same value when the spatial distribution remains the same as in the reference year T_0 (1989):

$$\%\widehat{Geo}_t = \%\Delta\hat{Y}_{FRt_{Adaptation}} - 100 \times \left(e^{W_{iT_0}{}^{T}\Delta X_{it_{Adaptation}}\hat{\beta}} - 1\right) \quad (22)$$

where $\%\Delta\hat{Y}_{FRt_{Adaptation}}$ is the result of **Equation 12** in the case of full adaptation, $\Delta X_{it_{Adaptation}}$ is the matrix containing the differences in the value of the weather variables found in **Equation 6** in the Methods for each department i between the year t and the reference year T_0 and where W_{iT_0} represents the vector containing the share of the national apple area tied to each department i in year T_0 (1989). This method is conceptually analogical to the Paasche Reallocation Index used in Beddow and Pardey[17] to measure the amount by which agricultural output increases due to the change in the relative spatial allocation of production over a given period.

8. First stage AR (1) regression to account for the effect of biennial bearing

Biennial bearing (also called alternate bearing) is the natural tendency of fruit trees to alternate crop load from year to year for biophysiological reasons and is particularly present in apple trees. Although apple farmers manage to reduce the impact of biennial bearing on their crop load through the use of both mechanical and chemical thinning techniques, annual yields still end up affected. For this reason, we estimate 32 department-specific AR(1) regressions using the maximum likelihood estimator in order to capture the inter-annual effect of biennial bearing on apple yields. We estimate these regressions on our yield data taken from the French Ministry of Agriculture's SAA (*Statistique agricole annuelle*) after having removed department-specific quadratic trends in order to make the data stationary. After having estimated the AR(1) regressions, we reconstruct yields net of the effect of biennial bearing by adding the residual of the AR(1) regression (i.e. the share of the detrended yield variation that is not due to biennial bearing) to each year's expected yield based on the quadratic time trend, which captures time-dependent yield variations caused by both climate trends and agronomic improvements. The detailed steps can be seen here below:

Step 1:

$$y_{it} = a_i t + b_i t^2 + \varepsilon_{it} \quad (23)$$

where y_{it} are department-specific annual yields (in tons/ha) and t is the number of years since the beginning of the sample period (1989).

Step 2:

$$\hat{\varepsilon}_{it} = \theta\hat{\varepsilon}_{it-1} + \mu_{it} \quad (24)$$

where $\hat{\varepsilon}_{it}$ and $\hat{\varepsilon}_{it-1}$ are the fitted residuals from **Equation 23** at year t and t-1 respectively and where θ represents the inter-annual dependency of yields, which is expected to be negative as a result of biennial bearing.

Step 3:

$$\hat{y}_{it} = \hat{a}_i t + \hat{b}_i t^2 + \hat{\mu}_{it} \ (25)$$

where $\hat{y}_{it}$ are predicted yields after adjustment for biennial bearing (the yields used in our analysis) and where $\hat{\mu}_{it}$ are the fitted residuals from **Equation 24**.

9. Climate adaptation in a non-stationary setting

In the Methods section, we define the effect of climate adaptation on expected yields for any given weather outcome as being the difference between **Equations 6 & 7** in the Methods, namely:

$$Adaptation = \beta_3[(x_{it} - \mu_{it})^2 - (x_{it} - \mu_{i0})^2] \ (26)$$

Considering that any weather outcome can be expressed as a combination of the prevailing climate, which corresponds to a 20-year moving average, and a random weather shock according to the formula $x_{it} = \mu_{it} + \varepsilon_{it}$, the expression defining adaptation then becomes:

$$Adaptation = \beta_3[\varepsilon_{it}^2 - (\mu_{it} + \varepsilon_{it} - \mu_{i0})^2] \ (27)$$

Defining $\Delta\mu_{it} = \mu_{it} - \mu_{i0}$ as the change in climate over the time period considered, we then obtain the following expression:

$$Adaptation = \beta_3[\varepsilon_{it}^2 - (\Delta\mu_{it}^2 + 2\Delta\mu_{it}\varepsilon_{it} + \varepsilon_{it}^2)] \ (28)$$

Rearranging the terms, this gives the final expression:

$$Adaptation = -\beta_3\Delta\mu_{it}^2 - 2\beta_3\Delta\mu_{it}\varepsilon_{it} \ (29)$$

where $-\beta_3\Delta\mu_{it}^2$ respresents the value of the climate penalty for a change in weather that matches the change in climate μ_{it}, which corresponds to the standard definition of climate adaptation according to Mérel and Gammans[8], and where $-2\beta_3\Delta\mu_{it}\varepsilon_{it}$ represents the effect of the random shock in weather happening at the new climate μ_{it}. Under a warming climate, the first term (i.e. the climate adaptation term) is always positive given that β_3 always takes on a negative value in the presence of adaptation. Regarding the second term, the sign of the shock ε_{it} determines its effect on the value of the yield improvement made possible by climate adaptation, with positive shocks raising its value and negative ones diminishing it. This definition of climate adaptation is more realistic than the standard one in which only the first term ($-\beta_3\Delta\mu_{it}^2$) is considered, since in real life growing systems almost never face weather outcomes that exactly match the local climate to which they are adapted ($x_{it} = \mu_{it}$). **Figure S3** located below graphically shows the difference between perfect climate adaptation and climate adaptation that takes into account random deviations in weather around the prevailing climate.

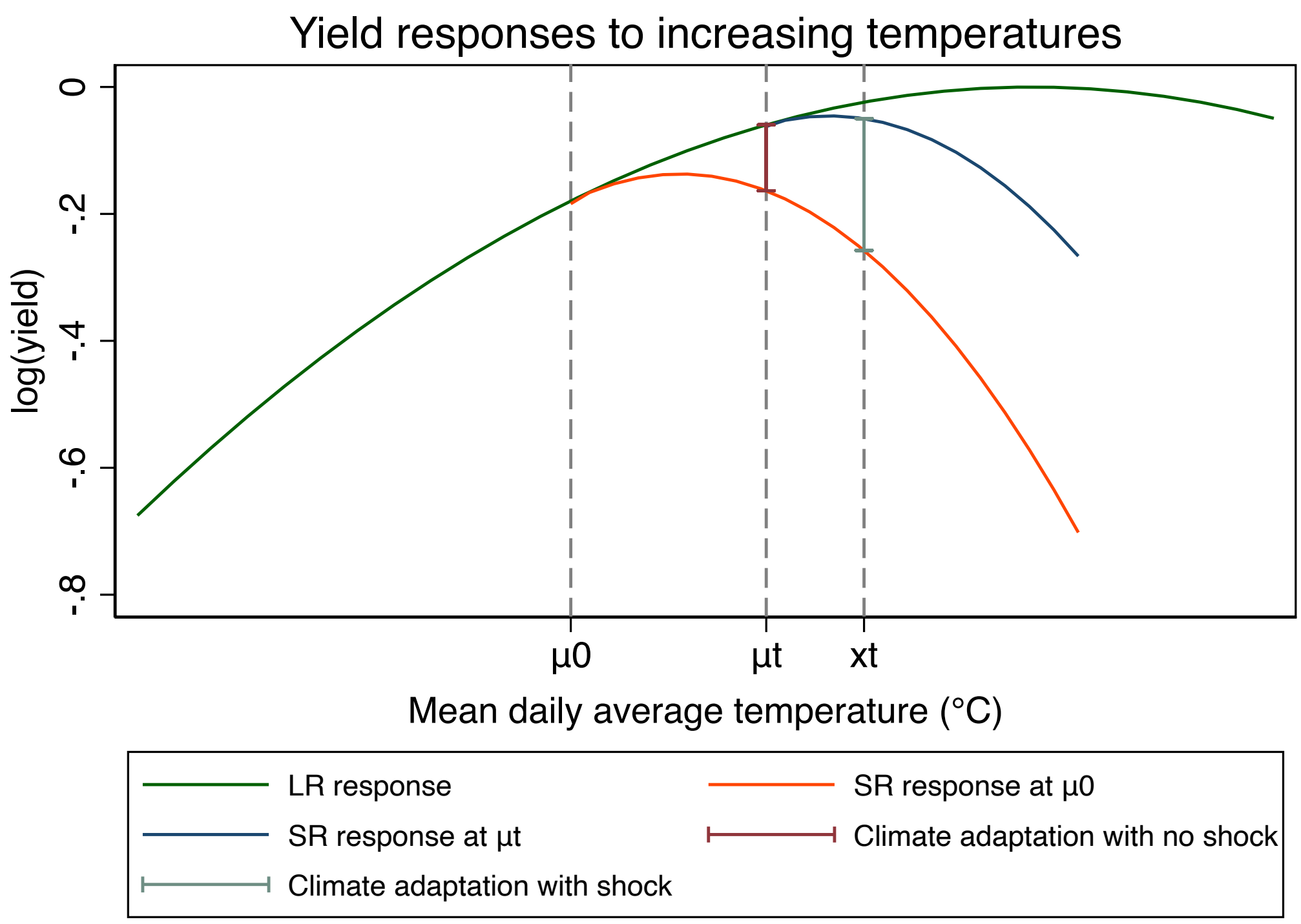

Figure S3: Climate adaptation in the context of a warming climate. Yield responses are for the blooming phase and are derived from Table S1's coefficients associated with the main version of the regression model.

10. Deriving the value of long-run and short-run temperature optima

To obtain the empirical temperature optima displayed in **Table S8** of the Supplementary Material and expressed as mean daily maximum temperatures, it is first necessary to calculate the optima in the form of mean daily average temperatures using the estimates of the model coefficients, as shown here below:

For the long run:

$$\frac{\partial \hat{y}_{LR}}{\partial x} = \hat{\beta}_1 + 2\hat{\beta}_2 x = 0$$

$$x^*_{LR} = \frac{-\hat{\beta}_1}{2\hat{\beta}_2} \quad (30)$$

For the short run:

$$\frac{\partial \hat{y}_{SR}}{\partial x} = \hat{\beta}_1 + 2\hat{\beta}_2 x + 2\hat{\beta}_3 (x - \mu) = 0$$

$$x^*_{SR} = \frac{2\hat{\beta}_3 \mu - \hat{\beta}_1}{2(\hat{\beta}_2 + \hat{\beta}_3)} \quad (31)$$

Once these values have been obtained, a pooled simple linear regression is run on the entire historical sample to find the correlation between mean daily average temperatures and mean daily maximum temperatures, which had already been computed at the departmental level. The value of x_{LR}^{*} or x_{SR}^{*} is then fitted to the regression to obtain the expected mean daily maximum temperature that correlates with the optimum previously found. For long-run optima, the temperatures used in the regression are 20-year moving averages (i.e. local climates), whereas they consist in yearly values (i.e. weather realizations) for short-run optima:

For the long run:

$$\mu(Tmax)_{it} = \delta + \beta\mu(Tmean)_{it} + \varepsilon_{it} \ (32)$$

For the short run:

$$x(Tmax)_{it} = \delta + \beta x(Tmean)_{it} + \varepsilon_{it} \ (33)$$

11. Clarification on the position of short-run temperature optima relative to local means and long-run optima

To illustrate where the optimal short-run temperature lies when the local climate is colder than the long-run optimum – as in the case of all departments in the blooming phase according to the model's estimates –, the first step consists in defining the local climate with respect to the long-run optimum for this case:

$$\mu = \frac{-\beta_1}{2\beta_2} - \gamma$$

$$where\ \gamma > 0$$

This expression is then inserted into **Equation 31** to obtain an expression of the short-run optimum conditional on the position of the local climate relative to the long-run optimum:

$$x_{SR}^{*} = \frac{2\beta_3(\frac{-\beta_1}{2\beta_2} - \gamma) - \beta_1}{2(\beta_2 + \beta_3)}$$

$$x_{SR}^{*} = \frac{-\beta_1}{2\beta_2} - \frac{\beta_3}{(\beta_2 + \beta_3)}\gamma, (34)$$

$$where\ 0 < \frac{\beta_3}{(\beta_2 + \beta_3)} < 1\ whenever\ \beta_2 < 0\ and\ \beta_3 < 0, thus\ implying\ that\ x_{SR}^{*} > \mu$$

The results from **Equation 34** demonstrate that whenever the temperature-yield relationship is concave and characterized by the existence of long-run adaptation, the short-term optimal temperature will always be higher than the local mean in cases where the local climate is colder than the long-run optimal climate. This also implies that in such locations, small positive temperature shocks ($\varepsilon < x_{SR}^{*} - \mu$) will lead to yield gains rather than yield losses, something consistent with plants' usual response to sudden temperature increases in sub-optimal climates.
